# Anwendung von Teduglutid bei Kurzdarmsyndrom im Säuglings-, Kindes- und Jugendalter: Positionspapier der Arbeitsgruppe „Chronisches Darmversagen“ der Gesellschaft für Pädiatrische Gastroenterologie und Ernährung (GPGE)

**DOI:** 10.1055/a-2757-3525

**Published:** 2026-02-11

**Authors:** Carsten Posovszky, Jan de Laffolie, Stephan Henning, Antje Ballauf, Jens Berrang, Victor Bildheim, Gunter Burmester, Andreas Entenmann, Gunter Matthias Christian Flemming, Judith Garino, Sebastian Gillmeister, Kathrin Krohn, Corinne Legeret, Eberhard Lurz, Steffen Reinsch, Franziska Righini-Grunder, Aline Rückel, Martina Kohl-Sobania, Johannes Hilberath

**Affiliations:** 1 30995 Gastroenterologie und Ernährung, Universitäts-Kinderspital Zürich, Zürich, Switzerland; 2 27197 Klinik für Kinder- und Jugendmedizin, University Ulm Medical Centre, Ulm, Germany; 3 60633 Paediatrics, Justus Liebig Universitat Giessen Fachbereich Medizin, Giessen, Germany; 4 Klinik für Pädiatrie mit Schwerpunkt Gastroenterologie, Nephrologie und Stoffwechselmedizin, Charite-Universitätsmedizin Berlin, Campus Virchow Klinikum, Berlin, Germany; 5 27664 Zentrum für Kinder- und Jugendmedizin, HELIOS Klinikum Krefeld, Krefeld, Germany; 6 39743 Westfälisches Kinderzentrum, Abteilung für Kindergastroenterologie, Klinikum Dortmund gGmbH, Dortmund, Germany; 7 9142 St. Josef-Hospital Bochum, Universitätsklinik für Kinder- und Jugendmedizin, Abteilung für Pädiatrische Gastroenterologie, Ruhr-Universität Bochum, Bochum, Germany; 8 155859 Gastroenterologie, AKK Altonaer Kinderkrankenhaus gGmbH, Hamburg, Germany; 9 27255 Universitätsklinikum Innsbruck, Department für Pädiatrie I, Universität Innsbruck, Innsbruck, Austria; 10 39066 Klinik für Kinder- und Jugendmedizin, Universitätsklinikum Leipzig, Leipzig, Germany; 11 Garino Medical Consulting, Nidda, Germany; 12 235861 Universitätsklinikum Ulm Klinik für Kinder- und Jugendmedizin, Ulm, Germany; 13 9183 Integriertes Sozialpädiatrisches Zentrum im Dr. von Haunerschen Kinderspital, Ludwig-Maximilians-Universität München, München, Germany; 14 30280 Pädiatrische Gastroenterologie, Hepatologie & Ernährung, Universitäts-Kinderspital beider Basel, Basel, Switzerland; 15 Pädiatrische Gastroenterologie und Hepatologie, Universitätsklinikum München, Dr. von Haunersches Kinderspital, München, Germany; 16 39065 Klinik für Kinder- und Jugendmedizin, Pädiatrische Gastroenterologie, Hepatologie und Endoskopie, Universitätsklinikum Jena, Jena, Germany; 17 Abteilung für Gastroenterologie, Hepatologie und Ernährung, Kinderspital Zentralschweitz, Luzern, Switzerland; 18 155853 Pädiatrische Gastroenterologie, Universitätsklinikum Erlangen Kinder- und Jugendklinik, Erlangen, Germany; 19 54186 Klinik für Kinder- und Jugendmedizin, Universitätsklinikum Schleswig-Holstein, Kiel, Germany; 20 219612 Abteilung Kinderheilkunde I, Pädiatrische Gastroenterologie und Hepatologie, Universitätsklinikum Tübingen Klinik für Kinder- und Jugendmedizin, Tübingen, Germany

**Keywords:** Kurzdarmsyndrom, chronisches Darmversagen, parenterale Ernährung, intestinale Rehabilitation, Glukagon-like peptide-2, Teduglutid, short bowel syndrome, chronic intestinal failure, parenteral support, intestinal rehabilitation, glucagon-like peptide-2, Teduglutide

## Abstract

Das pädiatrische Kurzdarmsyndrom (KDS) mit chronischem Darmversagen (CDV) ist eine seltene, komplexe und potenziell lebensbedrohliche Erkrankung. Die Hauptursachen im Kindesalter sind nekrotisierende Enterokolitis, Volvulus, angeborene Fehlbildungen und andere Enteropathien. Die resultierende verminderte intestinale Absorptionskapazität macht in vielen Fällen eine parenterale Ernährung (PE) erforderlich. Diese ist jedoch mit Komplikationen wie Katheter-Infektionen, Leber-Erkrankungen und Thrombosen sowie mit erheblichen Einschränkungen der Lebensqualität verbunden. Ziel der multiprofessionellen Behandlung ist es deshalb, eine enterale Autonomie anzustreben, Komplikationen zu vermeiden und die Lebensqualität zu verbessern. Seit der Einführung von Teduglutid, einem GLP-2-Analogon (GLP-2: Glucagon-like peptide-2), steht eine zusätzliche Therapie-Option zur Verfügung, die die Flüssigkeits- und Nährstoff-Aufnahme verbessern und eine Reduktion des parenteralen Unterstützungsbedarfs – bis hin zur partiellen oder vollständigen Entwöhnung – ermöglichen kann. Dieses Positionspapier fasst die bisherige Evidenz zur Wirksamkeit und Sicherheit von Teduglutid bei Kindern zusammen und gibt praxisorientierte Empfehlungen der Expert*innengruppe zu folgenden Aspekten: Definition relevanter Behandlungsziele (z.B. infusionsfreie Tage, enterale Autonomie), Kriterien für die Indikationsstellung, Einordnung in den Behandlungsalgorithmus, Anforderungen an Aufklärung, Betreuung, Monitoring und Verlaufskontrollen. Damit soll ein strukturierter Handlungsrahmen für den klinischen Einsatz von Teduglutid im Kindes- und Jugendalter geschaffen werden.

## Abstract (en)

Paediatric short bowel syndrome (SBS) with chronic intestinal failure (IF) is a rare, complex and potentially life-threatening condition. The main causes in children are necrotizing enterocolitis, volvulus, congenital malformations, and other enteropathies. The resulting reduced intestinal absorption capacity often requires parenteral nutrition (PN), which is associated with complications such as catheter infections, liver disease, and thrombosis, as well as significant limitations in quality of life. The goal of multidisciplinary treatment is to achieve enteral autonomy, prevent complications, and improve quality of life. With the introduction of the glucagon-like peptide-2 analogue (GLP-2-analogue) teduglutide, an additional therapeutic option is available that can improve fluid and nutrient absorption and reduce the need for parenteral support, up to partial or complete weaning. This position paper summarizes the current evidence on the efficacy and safety of teduglutide in children and provides practice-oriented recommendations from the expert group on the following aspects: Definition of relevant treatment goals (e.g., infusion-free days, enteral autonomy), criteria for indication, classification in the treatment algorithm, requirements for education, care, monitoring, and follow-up. The aim is to create a structured framework for the clinical use of teduglutide in children and adolescents.

## Einleitung

Die Gesellschaft für Pädiatrische Gastroenterologie und Ernährung (GPGE) und ihre Arbeitsgruppe „Chronisches Darmversagen“ haben zuletzt im März 2018 zur Therapie mit Teduglutid bei Kindern und Jugendlichen mit Kurzdarmsyndrom (KDS) Stellung genommen. Da zwischenzeitlich weitere Daten zur Sicherheit und Effektivität veröffentlicht wurden und die Zulassung auch auf das Säuglingsalter erweitert wurde, ist eine Aktualisierung erforderlich.

Das KDS mit chronischem Darmversagen (CDV) ist eine seltene, komplexe und potenziell lebensbedrohliche Erkrankung, die mit erheblichen Einschränkungen im Alltag der Patient*innen verbunden ist. Bei Kindern und Jugendlichen führt das Fehlen relevanter Darmanteile meist durch eine nekrotisierende Enterokolitis, einen Volvulus oder angeborene Fehlbildungen des Darms, wie zum Beispiel der intestinalen Atresie oder einer Gastroschisis, zu einem KDS 1 . Am häufigsten sind daher Neugeborene, Säuglinge und Kleinkinder betroffen.

Das CDV bei KDS ist gekennzeichnet durch eine unzureichende Resorption von Nährstoffen, Elektrolyten und Flüssigkeiten des verbleibenden Darms. Betroffene sind deshalb auch längerfristig auf eine vollständige oder teilweise heimparenterale Ernährung und Flüssigkeitszufuhr über einen permanent implantierten zentralvenösen Katheter angewiesen 2 3 .

Morbiditäts- bzw. mortalitätsrelevante Komplikationen, wie z.B. rezidivierende Infektionen zentralvenöser Katheter, thrombotische Gefäßverschlüsse, der progrediente Verlust von Gefäß-Zugangsmöglichkeiten sowie Wachstumsretardierung und organspezifische Komplikationen wie Nephropathie, Osteopathie oder Hepatopathie (Intestinal Failure Associated Liver Disease; IFALD) können auftreten.

Teduglutid ist ein Analogon des Glucagon-like Peptids-2 mit verlängerter Halbwertszeit. Es vermittelt Signale, die unter anderem eine Verlangsamung der Magen-Entleerung, eine Steigerung der Darmzottenhöhe und Kryptentiefe sowie der Gesamtmasse der Schleimhaut bewirken und somit zu einer Anregung der intestinalen und portalen Durchblutung führen. Darüber hinaus verbessert Teduglutid auch die epitheliale Barrierefunktion, indem es die Funktion der parazellulären Poren und Tight Junctions optimiert 4 . Diese Veränderungen steigern schließlich die Aufnahme von Flüssigkeit, Elektrolyten und Nährstoffen 5 6 7 8 .

Die Zulassung für Revestive (Wirkstoff: Teduglutid) zur Behandlung von erwachsenen Patient*innen mit KDS wurde am 30.08.2012 durch die Europäische Kommission erteilt.

In der Folge hat die S3-Leitlinie der Deutschen Gesellschaft für Ernährungsmedizin (DGEM) eine Therapie mit Teduglutid bei erwachsenen Patient*innen mit stabil infusionspflichtigem Darmversagen empfohlen, um infusionsfreie Tage zu gewinnen (Empfehlung 36) 2 .

Im Juli 2016 wurde Teduglutid (Revestive) in Deutschland für die Behandlung des KDS bei Kindern und Jugendlichen ab einem Alter von einem Jahr zugelassen. Grundlage hierfür war eine vergleichende, nicht randomisierte, offene klinische Zulassungsstudie über einen Zeitraum von 12 Wochen bei Kindern, auf deren Basis der Gemeinsame Bundesausschuss (G-BA) am 01.11.2016 eine Nutzenbewertung vornahm. Im Unterschied zur Nutzenbewertung bei Erwachsenen, die auf 2 randomisierten, placebokontrollierten, verblindeten Studien beruhte, konnte für die pädiatrische Indikation aufgrund der limitierten Evidenz kein Zusatznutzen belegt werden. Im Jahr 2023 wurde die Zulassung auf jüngere Patient*innen erweitert, sodass Revestive nun ab einem für Frühgeburtlichkeit korrigierten Alter von 4 Monaten bei Kindern mit Kurzdarmsyndrom eingesetzt werden kann. Vor diesem Hintergrund erscheint es umso wichtiger, die vorhandenen Daten kritisch einzuordnen und durch ein Expert*innen-Positionspapier praxisorientierte Empfehlungen für die Indikationsstellung, das Monitoring und die Therapieziel-Definition im Kindes- und Jugendalter bereitzustellen.

## Methodik

Es erfolgte eine Literaturrecherche in Pubmed unter den Stichwörtern „teduglutide“ und „pediatric short bowel“. Diese ergab 60 Treffer (Abfrage zuletzt am 25.03.2025). Es wurden 45 Artikel berücksichtigt (Originalarbeiten, Reviews und Positionspapiere); nicht humane Studien hingegen wurden ausgeschlossen. Darüber hinaus wurden weitere Publikationen per Handsuche hinzugefügt, darunter deutschsprachige Publikationen, AWMF-Leitlinien und Zulassungsstudien bei Erwachsenen. Das präliminäre Manuskript und die Empfehlungen wurden am 30. Januar 2025 in der Sitzung der Arbeitsgruppe (AG) „Chronisches Darmversagen“ der Gesellschaft für Pädiatrische Gastroenterologie und Ernährung (GPGE) vorgestellt und diskutiert. Entsprechende Empfehlungen wurden anschließend in einer ersten Delphi-Befragung vom 25.3.2025 bis zum 04.04.2025 abgestimmt. Dabei wurden die Interessenkonflikte der einzelnen AG-Mitglieder in Bezug auf die jeweilige Empfehlung abgefragt und die Ergebnisse mit und ohne Interessenkonflikte berücksichtigt. Es ergaben sich keine Unterschiede im Abstimmungsverhalten zwischen Mitgliedern mit und ohne Interessenkonflikt. Als Interessenkonflikte wurden Berater- und Referenten-Tätigkeiten sowie Zuwendungen für wissenschaftliche Projekte im Zusammenhang mit der Behandlung des Kurzdarmsyndroms gewertet. Zu insgesamt 10 Unterpunkten der 4 Empfehlungen gab es jeweils ein ablehnendes Votum und alternative Formulierungsvorschläge. Über diese wurde in einer 2. Delphi-Befragung vom 06.04.2025 bis zum 13.04.2025 und in einer 3. Delphi-Umfrage vom 16.04.2025 bis 25.04.2025 abgestimmt. Jedes Mitglied der AG war stimmberechtigt. Insgesamt 18 Expertinnen und Experten aus der AG haben am Delphi-Prozess teilgenommen. Als „Konsens“ wurde eine Zustimmung von 75–95% definiert, als „starker Konsens“ eine Zustimmung >95%. Es handelt sich um ein Expert*innen-Positionspapier, das zur Orientierung die vorhandene, wenn auch limitierte Evidenz aufführt. Die Evidenz wurde gemäß dem „Oxford Level of Evidence“ (2011) in die Klassen I–V eingestuft, und die Empfehlungsstärke als „soll“ (A), „sollte“ (B) oder „kann“ (C) angegeben. Das finale Manuskript wurde im E-Mail-Umlaufverfahren von allen Autor*innen angenommen. Der hohe Empfehlungsgrad der Empfehlungen – trotz niedriger Evidenz – ergibt sich aus der Patientenselektion in den Zulassungsstudien (Ein- und Ausschlusskriterien) und den berichteten Nebenwirkungen 9 10 11 12 . In den Zulassungsstudien wurden unter anderem Patient*innen mit aktiver entzündlicher Darmerkrankung, Motilitätsstörungen, PE-bedingter Hospitalisierung innerhalb der letzten 3 Monate oder Patient*innen, bei denen angenommen wurde, dass ihre Ernährung nicht steigerbar ist, ausgeschlossen 9 10 . Der starke Empfehlungsgrad dient vor allem auch der Patientensicherheit.

## Evidenzlage zur Wirksamkeit und Sicherheit von Teduglutid bei Kurzdarmsyndrom mit chronischem Darmversagen

Die Wirksamkeit und Sicherheit der täglichen subkutanen Gabe von Teduglutid (0,05 oder 0,1mg pro Kilogramm Körpergewicht) zur Behandlung von stabil infusionspflichtigen Erwachsenen mit Kurzdarmsyndrom wurde in 2 randomisierten, placebokontrollierten, doppelblinden, parallelen, multizentrischen und internationalen klinischen Studien der Phase III untersucht (n=169; davon n=78 mit 0,05mg/kg und n=32 mit 0,1mg/kg/d und n=59 mit Placebo) (CL0600–004 und CL0600–020, niedriges Verzerrungsrisiko) 13 14 . In der 24-wöchigen klinischen Studie (CL0600–004, NCT00172185, n=83) führte Teduglutid in einer Dosierung von 0,05mg/kg/d (n=35) im Gegensatz zu 0,1mg/kg/d (n=32) gegenüber Placebo (n=16) signifikant häufiger zu einer ≥ 20%igen Reduktion der wöchentlichen Infusionsmenge nach 20 bzw. 24 Wochen 13 . Auch in einer weiteren klinischen Studie (CL0600–020, NCT00798967, n=86) (Studienname: STEPS) führte die Behandlung mit 0,05mg Teduglutid bei 63% (n=27/43) im Vergleich zur Kontrollbehandlung mit 30% (n=13/43) nach 24 Wochen signifikant häufiger zu einer > 20%igen Reduktion der Infusionsmenge (p=0.002). Dies war verbunden mit einer statistisch signifikanten Volumenreduktion der parenteralen Ernährung (p < 0,001) 14 . Außerdem fand sich ein statistisch signifikanter Gewinn von mindestens einem infusionsfreien Tag (21/39 [54%] gegenüber Placebo (n=9/39 [23%]; p = 0,005) 14 . In der 24-monatigen Open-label-Verlängerungsstudie (STEPS-2) erreichten in der Intention-to-treat-Population (n=88) 89% der durchgängig mit Teduglutid behandelten Patient*innen (n=33/37), 46% der initial 24 Wochen mit einem Placebo behandelten und (später) auf Teduglutid umgestellten Patient*innen (n=18/39) sowie 50% der eingeschlossenen, aber nicht mehr randomisierten und nun open-label mit Teduglutid behandelten (n=6/12) Patient*innen ein klinisches Ansprechen, definiert als eine ≥ 20%ige Reduktion des Infusionsvolumens gegenüber dem Ausgangswert 15 . Insgesamt erreichten 13 von 65 Patient*innen (20%) bis zum Studienende eine enterale Autonomie, davon 10 aus der Untergruppe, die durchgehend mit Teduglutid behandelt wurden (n=10/30, 33%). Teduglutid (Revestive) wurde am 30. August 2012 von der Europäischen Kommission zugelassen und am 1. September 2014 in Deutschland zur Behandlung von Erwachsenen eingeführt.

An einer klinischen Open-label-Studie nahmen 42 pädiatrische Kurzdarm-Patient*innen im Alter von 1–14 Jahren teil, bei denen der Aufbau enteraler Ernährung nur minimale oder keine Fortschritte machte und bei denen noch mindestens 30% der Tagesmenge an Flüssigkeit oder Kalorien parenteral verabreicht wurden 9 . Sie erhielten über 12 Wochen 0,0125 (n=8); 0,025 (n=14) und 0,05 (n=15) mg/kgKG/d Teduglutid, subkutan oder als Standard-Behandlung (n=5) (NCT01952080, hohes Verzerrungspotenzial auf Studien- und Endpunkt-Ebene). Im Vergleich zur Standard-Therapie gaben die mit Teduglutid behandelten Teilnehmer*innen häufiger Erbrechen, Übelkeit, Kopfschmerzen und Müdigkeit sowie Hämatome an der Injektionsstelle an. Kein Patient brach die Studie jedoch wegen eines unerwünschten Ereignisses ab. Nach 12 Wochen zeigte sich sowohl bei einer Dosierung von 0,025 als auch von 0,05mg/kgKG/d ein Trend zur Reduktion der parenteralen Ernährung um 25 bzw. 41% des Gesamtvolumens und um 45% bzw. 52% der Kalorien im Median. In den Gruppen mit Standard-Behandlung und 0,00125mg/kg/d Teduglutid waren hingegen keine Veränderungen festzustellen 9 . Insgesamt konnten 4 Patient*innen (10,8%) von der parenteralen Ernährung entwöhnt werden, 3 in der Gruppe mit 0,05mg/kg/Tag und ein Patient in der Gruppe mit 0,025mg/kg/Tag.

Danach wurde eine 24-wöchige Phase-III-Studie mit 2 randomisierten, doppelblinden Teduglutid-Dosisgruppen mit 0,025 (n=24) und 0,05mg/kgKG/d (n=26) und einem nicht verblindeten und nicht randomisiert zugeordneten Standard-Behandlungsarm (n=9) durchgeführt (NCT02682381) 10 . Es gab keine Unterschiede zu den Ein- und Ausschlusskriterien zur vorherigen Studie, und alle 59 eingeschlossenen Patient*innen haben die Studie wie geplant abgeschlossen. Den primären Endpunkt einer ≥ 20%igen Reduktion der parenteralen Ernährung erreichten mit 54,2% (n=13) unter Dosierung mit 0,025mg/kgKG/d und 69,2% (n=18) unter Dosierung mit 0,05mg/kgKG/d signifikant mehr Patient*innen als in der Kontrollgruppe (11,1%, n=1). In beiden Teduglutid-Dosisgruppen wurden eine signifikante Reduktion des parenteralen Ernährungsvolumens und der parenteralen Kalorienaufnahme sowie eine Verminderung der Infusionsdauer beobachtet. Außerdem nahm die enterale Nahrungszufuhr zu. Insgesamt erreichten 5 Patient*innen (10%) eine enterale Autonomie, 2 unter der Dosierung von 0,025mg/kgKG/d und 3 unter 0,05mg/kgKG/d. Allerdings erreichten 3 der 5 Patient*innen die enterale Autonomie erst nach 12 Wochen Behandlung. Das Nebenwirkungsprofil war ähnlich den bereits für Erwachsene und Kinder publizierten Angaben. Unter anderem wurden Bauchschmerzen, Durchfall, Dehydrierung, Kopfschmerzen, Rhinitis und Transaminasen-Erhöhungen häufiger als im Standard-Behandlungsarm berichtet. In dieser 24-Wochen-Studie zeigten sich auch noch 4 Wochen nach Beendigung der Behandlung Effekte von Teduglutid auf die Kalorien- und Flüssigkeitszufuhr 10 .

Post-hoc-Analysen ergaben auch eine Verbesserung der Stuhlkonsistenz 16 .

In 2 offenen Phase-III-Studien und einer Langzeit-Folgestudie wurde die kurz- und langfristige Sicherheit und Wirksamkeit von Teduglutid (0,05mg/kgKG/d) bei Säuglingen und Kindern mit CDV untersucht. Es handelt sich um die Studien NCT03571516 (europäische 24-Wochen-Studie mit Säuglingen, die randomisiert Teduglutid [n=5] oder die Standard-Behandlung [n=5] erhielten); NCT02980666 (japanische 24-wöchige Studie mit Säuglingen [n=2] und Kindern [n=8], die alle Teduglutid erhielten) und deren 24-wöchige Verlängerungsstudie NCT03268811, mit Patient*innen, die NCT02980666 abgeschlossen hatten (somit konnten die Patient*innen insgesamt bis zu 48 Wochen lang behandelt werden) 17 18 . In die europäische Säuglingsstudie konnten nur Patient*innen mit parenteralem Flüssigkeits- und/oder Kalorienbedarf von mindestens 50% eingeschlossen werden, während die Grenze in der japanischen Studie mit 30% analog zu anderen pädiatrischen Studien lag. Bei einer insgesamt geringen Fallzahl von 7 Säuglingen (wovon bei Therapiebeginn 2 Säuglinge 4–6 Monate und 5 Säuglinge 6–12 Monate alt waren) und 8 Kindern, die Teduglutid über 24 Wochen erhielten, zeigte sich auch in diesen Studien eine Verträglichkeit und Sicherheit, die den Ergebnissen vorangegangener Publikationen entspricht. Hinsichtlich der Wirksamkeit konnte nach 24 Wochen bei 4 Säuglingen (57,1%) in der Teduglutid-Gruppe und für 2 Säuglinge (50%) sowie 4 Kinder (66,7%) in der Kontroll-Gruppe eine Reduktion der parenteralen Ernährung von ≥ 20% erreicht werden. Enterale Autonomie erreichten 2 Kinder (25%) nach 12 bzw. 28 Wochen Behandlung mit Teduglutid, jedoch keiner der in die Studie eingeschlossenen Säuglinge.

Erfahrungen aus der Praxis haben die Sicherheit und das positive Wirkungspotenzial von Teduglutid bei Kindern mit einer Reduktion des parenteralen Ernährungsbedarfs bestätigt 19 20 21 22 23 24 . Bisher wurden Behandlungsdaten von 13 Kindern aus Israel (im Median 6 Jahre alt bei Behandlungsbeginn), 7 Kindern aus der Slowakei (im Median 7,99 Jahre), 25 Kindern aus Frankreich (im Median 9,4 Jahre), 31 Kindern aus Spanien (im Median 2,3 Jahre) und 104 Kindern aus europäischen Zentren (im Median 6,7 Jahre) publiziert 20 21 22 23 24 . In der französischen Studie stieg der Plasma-Citrullin-Wert (ein indirekter Marker der Enterozytenmasse) von 14 μmol/l (IQR: 8–21) zu Studienbeginn auf 29 μmol/l (IQR: 17–54) in Woche 48 (P<0,001) 22 . Das Ausmaß des Ansprechens sowie das Zeitintervall der Wirkungsentfaltung sind sehr variabel 20 25 .

Prädiktive Faktoren für ein positives bzw. fehlendes Ansprechen konnten bis dato für die Praxis noch nicht verlässlich ermittelt werden. In der spanischen Kohorte konnte bei frühem Behandlungsbeginn (im Mittel im Alter von 3 Jahren) das Ziel der 20%igen Flüssigkeitsreduktion statistisch signifikant öfter erreicht werden als bei späterem Beginn (im Mittel im Alter von 6,3 Jahren), jedoch traf dies nicht für eine 20%ige Kalorienreduktion zu 21 .

Das Sicherheitsprofil von Teduglutid bei Kindern und Jugendlichen ist insgesamt vergleichbar mit den Beschreibungen bei Erwachsenen. Die folgenden Ereignisse wurden sehr häufig beobachtet: Atemwegsinfektionen, Kopfschmerzen, abdominale Distension und Schmerzen, Übelkeit, Erbrechen, Reaktionen an der Injektionsstelle sowie gastrointestinale Stoma-Komplikationen. Eine Analyse der gepoolten Daten aus 4 klinischen Studien ergab als häufigste unerwünschte Ereignisse Erbrechen, Fieber, obere Atemwegsinfekte und Husten sowie 3 schwere Ereignisse (Ileus, D-Laktat-Azidose, intestinale Obstruktion durch festen Stuhlgang) 11 . Auch in einer Anwendungsbeobachtung von 25 Patient*innen über 48 Wochen zeigten sich keine anderen behandlungsbedingten Nebenwirkungen 22 . Angehörige von Gesundheitsberufen sind aufgefordert, jeden Verdacht auf eine Nebenwirkung zu melden ( www.bfarm.de ).

Für Säuglinge unter 4 Monaten liegen noch keine Daten vor. Die Datenlage zur Langzeit-Sicherheit bei Säuglingen, Kindern und Jugendlichen ist begrenzt. In einer Kanzerogenitätsstudie an Ratten wurden gutartige Tumoren im Dünndarm und in den extrahepatischen Gallengängen festgestellt. Diese Befunde konnten in klinischen Humanstudien, die länger als ein Jahr dauerten, nicht bestätigt werden.

Bei erwachsenen Patient*innen ist das Auftreten von adenomatösen Dick- und Dünndarm-Polypen während einer Teduglutid-Therapie beschrieben 26 27 . Ebenso existieren vereinzelt Fallberichte zu Polypen bei pädiatrischen Patient*innen 28 29 . Die Inzidenz des Auftretens von intestinalen Polypen bei Kindern unter Langzeit-Therapie mit Teduglutid ist bislang nicht bekannt.

## Behandlungsziele

Behandlungsziele für Kinder und Jugendliche mit CDV sind eine individuell optimierte Darmfunktion, mit dem Ziel, infusionsfreie Tage zu ermöglichen und – soweit erreichbar – eine vollständige enterale Autonomie zu erlangen. Aus Sicht der Autor*innen ist die Zunahme infusionsfreier Zeiträume bzw. Tage besonders relevant und geht über den Nutzen einer alleinigen Reduktion des täglichen parenteralen Supports hinaus. Insbesondere bei Schulkindern und Jugendlichen verbessert sie, in der Erfahrung der Autor*innen, die Möglichkeit zur sozialen Teilhabe und Unabhängigkeit, was sich positiv auf die Lebensqualität sowie das Wohlbefinden der Familie auswirken kann 30 31 . Darüber hinaus könnten durch die Verringerung der PE-Tage auch therapieassoziierte Nebenwirkungen und Komplikationen reduziert werden. Gleichzeitig muss die adäquate Zufuhr von Nährstoffen und Flüssigkeit für den wachsenden Organismus jederzeit sichergestellt werden.

Für die Gesamtprognose der betroffenen Patient*innen mit CDV ist eine optimale intestinale Rehabilitation entscheidend. Hier greifen interdisziplinäre und multiprofessionelle Behandlungsstrategien, unter anderem aus der Neonatologie, Kinderchirurgie, Kinder-Gastroenterologie, Fachpflege, Psychologie und Ernährungsmedizin synergistisch ineinander. Entsprechende multidisziplinäre intestinale Rehabilitationsprogramme erfolgen in Zentren und in Kooperation mit erfahrenen Behandler*innen. Sie reduzieren die Morbidität und Mortalität 32 . Darüber hinaus benötigen die Sorgeberechtigten und die Patient*innen eine kinderärztliche und pflegerische Vor-Ort-Betreuung, eine spezialisierte Ernährungsberatung und ggf. sozialmedizinische und psychologische Unterstützung. Die Lebensqualität bei CDV ist sowohl für die Patient*innen als auch für deren Familien signifikant reduziert 33 34 .

## Behandlung mit Teduglutid im Kontext des Kurzdarmsyndroms

**Abb. 1:**
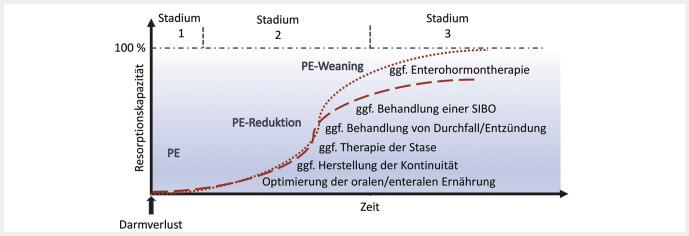
Intestinale Resorptionskapazität und parenteraler Ernährungssupport nach chirurgischem Darmverlust mit chronischem Darmversagen im zeitlichen Verlauf mit Maßnahmen der intestinalen Rehabilitation. In der akuten Phase des KDS nach Darmverlust (Stadium 1) liegt eine Hypersekretion vor, die etwa 1–6 Wochen dauert. Dann beginnt das Stadium 2 mit der Adaptation, das etwa 4 Wochen bis ein Jahr andauert. Anschließend folgt die Stabilisierung im Stadium 3, die etwa 4 Monate bis zu einem Jahr andauert. Zu den Maßnahmen der intestinalen Rehabilitation gehören unter anderem: 1) orale/enterale Ernährung und Flüssigkeitszufuhr mit Stimulation der Adaptation, Optimierung der Digestion und Absorption; 2) kontinuierlicher intestinaler Transport ohne Stase, z.B. durch chirurgische Behandlung von Stenosen oder Rückverlagerung von Stomata mit Wiederherstellung einer Darmkontinuität; 3) Therapie der (chologenen) Diarrhoe, Hypersekretion, beschleunigter Transitzeit oder Entzündung; 4) Behandlung der bakteriellen Fehlbesiedlung (SIBO) mit (nicht resorbierbaren) Antibiotika; 5) Enterohormon-Therapie mit GLP-2-Analoga, z.B. Teduglutid. Mit zunehmender intestinaler Resorptionskapazität sinkt der Bedarf an parenteralem Kalorien- und Flüssigkeitssupport (blauer Hintergrund). Idealerweise kann die Zufuhr von PE schrittweise reduziert werden, sodass PE-freie Tage möglich werden (PE-Weaning) und schließlich eine enterale Autonomie erreicht wird, bei der die Resorptionskapazität 100% des Nährstoff- und Flüssigkeitsbedarfs deckt (gepunktete Linie). Wenn trotz optimierter Behandlung eine unzureichende Resorptionskapazität besteht (gestrichelte Linie), könnte eine Behandlung mit dem Enterohormon Teduglutid zu einer weiteren Verbesserung der Resorptionskapazität beitragen und im optimalen Fall auch zur enteralen Autonomie führen.

**Abb. 2:**
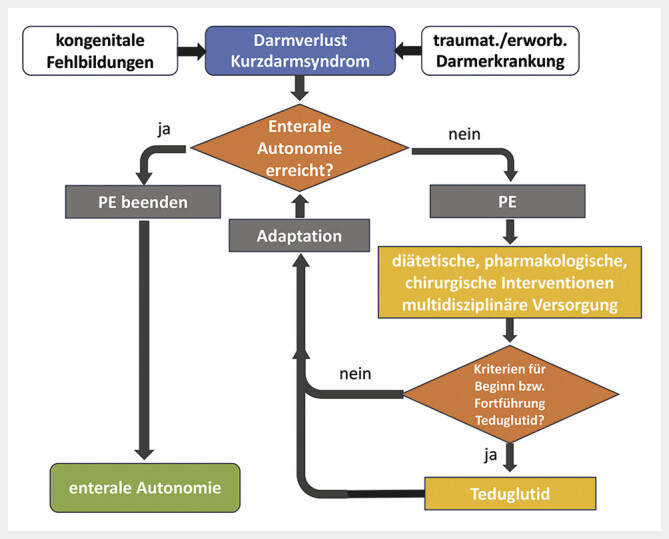
Behandlungsalgorithmus bei Kurzdarmsyndrom mit Darmversagen. Die Abbildung zeigt das therapeutische Vorgehen zur Förderung der Adaptation beim Kurzdarmsyndrom. Nach einem Darmverlust erfolgt zunächst eine Parenterale Ernährung (PE) und eine multidisziplinäre Versorgung mit individuellen Maßnahmen, um die Adaptation zu fördern. Bei erfolgreicher Adaptation wird die PE beendet und das Ziel der „enteralen Autonomie“ (grün) erreicht. Liegen die Kriterien für eine Behandlung mit Teduglutid vor (z.B. stagnierende Adaptation), wird diese initiiert, um das Erreichen einer enteralen Unabhängigkeit zu unterstützen. Die Behandlung mit Teduglutid wird solange fortgeführt, bis eine allgemeine Verbesserung des Zustands erreicht wird. In manchen Fällen ist trotz all dieser Maßnahmen eine langfristige, bedarfsgerechte PE erforderlich.

Teduglutid (Revestive) ist gemäß der Fachinformation für Patient*innen mit angeborenem oder erworbenem KDS, unabhängig von der Rest-Dünndarmlänge, zugelassen. Es kann zur Behandlung von Patient*innen ab einem korrigierten Gestationsalter von 4 Monaten angewendet werden. Nach einem chirurgischen Eingriff sollte zunächst eine Phase der intestinalen Adaptation abgewartet werden, und die Patient*innen sollten sich zudem in einer stabilen Phase der Darmrehabilitation befinden (Fachinformation Revestive 1,25mg, Stand: Juli 2024) ( Abb. 1 , Abb. 2 ).

Die empfohlene Dosis von Revestive bei Kindern und Jugendlichen (im Alter von korrigiert 4 Monaten bis 17 Jahren) beträgt 0,05mg pro kg/Körpergewicht und wird 1-mal täglich subkutan appliziert. Die rekonstituierte Revestive-Lösung ist laut Hersteller bei Raumtemperatur (25°C) bis maximal 24 Stunden chemisch und physikalisch stabil. Im Falle einer mäßigen bis schweren Nierenfunktionsstörung ist die Dosis anzupassen. Revestive wurde bei Patient*innen mit schwerer Leberfunktionsstörung nicht untersucht.

Die Expert*innengruppe sieht die Therapie mit Teduglutid als einen weiteren Bestandteil des Behandlungskonzepts für Kinder mit Kurzdarmsyndrom und chronischem Darmversagen (CDV). Anhand der vorliegenden Publikationen kann von einer Reduktion der parenteralen Zufuhr um ≥ 20% in 62–96% der behandelten Kinder mit CDV ausgegangen werden (Evidenzklasse 2) 10 19 20 22 24 35 . Das Ansprechen darüber hinaus ist variabel. Gemäß einer Beobachtungsstudie ist es bei Kindern mit längerem Restdünndarm, besserem Ernährungszustand und Kindern ohne Leber-Erkrankung besser 24 . In den klinischen Studien zeigte sich bei Patient*innen mit ≥ 50% Dickdarm in Kontinuität ein Trend zu einer größeren Anzahl von PE-freien Tagen pro Woche 36 . Die vollständige Entwöhnung von der PE war in der multizentrischen europäischen Kohorte mit einem geringeren Kalorienbedarf über die PE und einem höheren Citrullinwert zu Beginn der Teduglutid-Behandlung assoziiert 24 . Inwieweit sich das Ansprechen der Therapie mit Teduglutid auf die vor Therapiebeginn vereinbarten Ziele auswirkt, ist regelmäßig zu evaluieren (zu Beginn monatlich und im Verlauf vierteljährlich, siehe auch Monitoring). Bei fehlendem Ansprechen, unzureichender oraler bzw. enteraler Steigerungsmöglichkeit der Nahrungszufuhr oder schweren Nebenwirkungen ist diese auch wieder zu beenden.

In ihrem Positionspapier zum Langzeit-Management bei KDS nimmt die European Society of Paediatric Gastroenterology, Hepatology and Nutrition (ESPGHAN) in 4 Empfehlungen zur Behandlung der intestinalen Absorption mit Teduglutid Stellung. Diese 4 praktischen Punkte haben wir in unserem Positionspapier berücksichtigt. Dabei unterscheidet sich der Empfehlungsgrad bei der Indikation zur Teduglutid-Behandlung von unserer Empfehlung 1.I. Demgemäß „ *sollte* “ die Therapie mit Teduglutid bei stoffwechselstabilen Kindern und Jugendlichen mit Darmversagen bei Kurzdarmsyndrom in Betracht gezogen werden, wenn die intestinale Adaptation trotz angemessener Untersuchung und Behandlung durch ein spezialisiertes multidisziplinäres, pädiatrisches, intestinales Rehabilitationsteam stagniert 12 . Außerdem sollten Kinder mit limitierten venösen Gefäß-Zugangsmöglichkeiten oder Leber-Erkrankungen demnach bei der Indikationsstellung für Teduglutid besonders berücksichtigt werden 12 . Diese und andere Situationen, in denen der Einsatz von Teduglutid als besonders dringlich erachtet werden kann, haben wir unter 1.II als individuelle Nutzen-Risiko-Abwägung mit starkem Empfehlungsgrad zusammengefasst und die entsprechenden Kriterien im Hintergrundtext benannt (siehe unten).

Eine möglichst effiziente und somit sinnvolle Anwendung von Teduglutid als GLP-2-Analogon erfolgt unter optimierten und stabilen, individuellen Bedingungen ( Abb. 1 ). Eine Grundvoraussetzung im Behandlungskonzept ist, dass eine enterale bzw. orale Ernährung möglich und steigerbar ist. Vorrangig ist auch das Erreichen einer Kontinuität der Darm-Anatomie durch Rückverlagerung von Stomata oder Beseitigung von Stenosen, die zur Stase führen. Im Einzelfall sind auch Maßnahmen der rekonstruktiven oder darmverlängernden Chirurgie an einem erfahrenen Zentrum auszuschöpfen. Ebenso sind eine (chologene) Diarrhoe, intestinale Inflammation oder bakterielle Fehlbesiedelung primär zu behandeln ( Abb. 1 , Abb. 2 ). Es wird diskutiert, welche Kurzdarm-Situation am besten geeignet ist und ob Patienten, die kurz vor der Entwöhnung von der PE stehen, oder diejenigen, die am stärksten von der PE abhängig sind, behandelt werden sollten 37 . Im ersten Fall ist eine Behandlung mit Teduglutid eher von kurzer Dauer und eine enterale Autonomie das Ziel, während im zweiten Fall eine längerfristige Behandlung, mit dem Ziel, infusionsfreie Tage zu erreichen, im Vordergrund steht. Es gibt auch die Meinung, dass ein Mindestalter und eine Mindestdauer der parenteralen Ernährung (PE) von 3 Jahren erforderlich sind, um die Verzögerung und die Auswirkungen der physiologischen Darmanpassung nach einer umfangreichen Resektion zu berücksichtigen 22 .

Das Überleben der Patient*innen hängt maßgeblich von Komorbiditäten und Komplikationen ab, die mit dem CDV assoziiert sind. Dazu zählen katheterinduzierte Blutstrom-Infektionen, CDV-bedingte Leber-Erkrankungen, Thrombosen, Elektrolytstörungen und eine Mangelernährung. In den vergangenen Jahren konnte die Überlebensrate durch die Einführung multidisziplinärer Darm-Rehabilitationsprogramme, darmverlängernder Verfahren sowie durch die Verwendung neuer und verbesserter parenteraler Ernährung gesteigert werden. Zwar sind die Mortalitäts- und Transplantationsraten bei Kindern mit CDV zurückgegangen, jedoch hat sich der Anteil der Kinder, die eine enterale Autonomie erreichen, kaum verändert 38 . Ein erheblicher Teil von ihnen bleibt weiterhin auf parenterale Ernährung angewiesen 38 . Daraus ergibt sich die Notwendigkeit zur Erschließung neuer Strategien zur Förderung der enteralen Autonomie. Aufgrund der nachgewiesenen Sicherheit und Effektivität von Teduglutid, den Infusionsbedarf zu reduzieren und teilweise eine enterale Autonomie zu ermöglichen, stellt diese Therapie-Option einen möglichen Ansatz dar 35 37 . Allerdings liegt bisher keine Evidenz dafür vor, dass sich die Therapie mit Teduglutid günstig auf CDV-assoziierte Komorbiditäten und Komplikationen auswirkt 35 .

Vor diesem Hintergrund hat die Expert*innengruppe Kriterien formuliert, die eine Therapie-Entscheidung im Sinne einer Nutzen-Risiko-Abwägung unterstützen sollen.

Drohende oder bestehende Komplikationen, z.B. durch:

- Chronische mit dem CDV assoziierte Leberschädigung (IFALD)
- Multiple Gefäßverschlüsse mit sich entwickelndem oder drohendem Gefäß-Zugangsverlust für zentralvenöse Katheter
- Kleinwuchs oder Gedeihstörung
- Rezidivierende katheterassoziierte Sepsis-Episoden sowie daraus resultierende Katheter-Entfernungen und Neuanlagen

Für den Einsatz von Teduglutid vor Einleitung einer parenteralen Ernährung liegen bislang keine kontrollierten klinischen Studiendaten vor. Die verfügbare Evidenz bezieht sich ausschließlich auf Patient*innen, die bereits PE-abhängig sind. Ein präemptiver Einsatz kann daher derzeit nicht regelhaft empfohlen werden, auch wenn er gemäß der Fachinformation (Stand 7/2024) zulässig ist. In ausgewählten Einzelfällen könnte jedoch nach Einschätzung der Expert*innengruppe – gestützt durch eigene, noch nicht publizierte Erfahrungen – eine Therapie mit Teduglutid in Betracht gezogen werden: sowohl in einer frühen Phase, d.h. vor Erreichen des Adaptationsplateaus, als auch zur Vermeidung einer parenteralen Ernährung bei Patient*innen mit kritischer Darmfunktion, mit oder ohne KDS-assoziierten Komplikationen. Beispiele hierfür sind:

- drohende bzw. erneute parenterale Ernährung (z.B. nach Absetzen von Teduglutid) trotz Ausschöpfung KDS-spezifischer Maßnahmen, inklusive optimierter oral-enteraler Ernährung mit Gewichtsabnahme, Malnutrition, Gedeihstörung und metabolischer Entgleisung
- eine ungünstige bzw. drohende terminale venöse Gefäßsituation
- chronische, mit dem KDS assoziierte Hepatopathie (IFALD)
- individuelles Patient*innen-Umfeld, in dem eine heimparenterale Ernährung ein nicht zu vertretendes Risiko für lebensbedrohliche, katheterassoziierte Infektionen darstellt und auch nicht durch geeignete Unterstützungsmöglichkeiten kompensiert werden kann.

Wenn eine individuelle Therapie-Indikation gestellt wird, die von der Zulassung nicht abgedeckt ist, wird in diesen Einzelfällen die Beantragung der Kostenübernahme bei der Krankenkasse empfohlen. Patient*innenbezogene Kostenanalysen weisen darauf hin, dass erhebliche krankenhausbedingte Kosten im Zusammenhang mit Komplikationen durch das KDS und seine Behandlung entstehen. Hierzu gehören der Darmverschluss, katheterassoziierte Infektionen, das Anlegen von Ernährungsstomata und die Aufnahme auf die Intensivstation 39 . Demgegenüber stehen die Behandlungskosten mit Teduglutid. Inwiefern die Kosten der Therapie mit Teduglutid höher sind als die Kosten für die PE und das Management der Komplikationen, lässt sich nicht allgemein beantworten 39 . Wenn ein Kind jedoch vollständig von der PE entwöhnt wird, dürfte der Nutzen für das Kind durch den Wegfall des zentral-venösen Langzeit-Katheters und die Befreiung von einer hochtechnischen, teuren und komplikationsreichen Behandlung, bei nahezu normaler Lebenserwartung, die Kosten aufwiegen.

Es sind weitere Studien und ein internationaler Konsens erforderlich, um die jeweiligen Indikationen und den Zeitpunkt der Teduglutid-Behandlung festzulegen 40 .

### Aufklärung

Im Vorfeld ist eine offene Kommunikation und Beratung der Patient*innen bzw. ihrer Sorgeberechtigten über Nutzen, Nebenwirkungen und Risiken sowie über die erforderliche Therapietreue und die Kontrollen (vor Therapiebeginn und unter Therapie) notwendig. Die detaillierte Aufklärung der Sorgeberechtigten sowie der Kinder und Jugendlichen in altersgerechter Form sollte vor Beginn der Therapie mit Revestive schriftlich dokumentiert werden. Als Grundlage der Aufklärung soll die jeweilige aktuelle Fachinformation von Revestive (Teduglutid) verwendet werden. Darüber hinaus können auch andere Aufklärungsmaterialien herangezogen werden.

### Betreuung

Teduglutid stellt keine Alternative zu einer guten medizinischen Versorgung dar 12 . Deshalb sollte die Behandlung unter der Aufsicht von Ärzt*innen mit Erfahrung in der Behandlung von Kindern und Jugendlichen mit KDS begonnen werden. Das therapieeinleitende Behandlungsteam soll sich vor Therapiebeginn (insbesondere bei der Behandlung von Säuglingen bzw. sehr jungen Patient*innen) mit einer auf das Kurzdarmsyndrom spezialisierten Kinder-Gastroenterologie austauschen. Dabei sollten die Therapie-Voraussetzungen geprüft sowie der geeignete Zeitpunkt des Therapiebeginns, das Monitoring der Therapie und die Langzeit-Verlaufskontrollen besprochen werden. Hierzu gehören auch obere und untere Endoskopien sowie ggf. eine Bildgebung zur Tumorvorsorge und Erfassung von Komplikationen.

Kinder mit KDS, die mit einer heimparenteralen Ernährung entlassen werden, sollten von einer ausgewiesenen multidisziplinären Abteilung für die Rehabilitation bei Darmversagen betreut werden 12 . Zu den Mindestanforderungen einer multidisziplinären und multiprofessionellen Betreuung gehören ein/e Kinder-Gastroenterologe/in mit Ausbildung in klinischer Ernährung, ein/e Kinderchirurg/in, Fachpflegekraft, Ernährungsfachkraft und ein/e Apotheker/in mit Erfahrung in parenteraler Ernährung 12 . Die Betreuung durch multidisziplinäre Teams führt nachweislich zu einer Reduktion der Morbidität und Mortalität. Eine Meta-Analyse ergab, dass bei Patient*innen in einem intestinalen Rehabilitationsprogramm (n=130) im Vergleich zu historischen Kontrollen (n=103) weniger septische Episoden (0,3 vs. 0,5 Ereignisse pro Monat; p=0,01) auftraten und die Gesamtüberlebensrate der Patient*innen stieg (von 22% auf 42%) 32 .

## Praktisches Vorgehen bei Therapiebeginn und im Verlauf

Um die Therapieziele – PE-Reduktion, infusionsfreie Tage und gegebenenfalls enterale Autonomie – zu erreichen, ist es essenziell, dass bei Therapiebeginn mit Teduglutid eine orale oder enterale Ernährungszufuhr etabliert ist oder in der Folge erwartungsgemäß ausreichend gesteigert werden kann. Bei ausschließlich oral ernährten Kindern sind eine orale Aversion sowie Fütter- bzw. Essstörungen zu berücksichtigen.

### Stationärer Behandlungsbeginn mit Revestive

Bei Kleinkindern bis 6 Jahre kann ein stationärer Therapiestart in Erwägung gezogen werden und er sollte unter engmaschiger ambulanter Kontrolle erfolgen. Dies gilt ebenso bei komplexen Grunderkrankungen oder Begleiterkrankungen (z.B. Nierenfunktionsstörungen, kardialen Erkrankungen mit Herzinsuffizienz), Spritzenphobien, schwierigen familiären Situationen sowie Problemen mit der anfänglichen Compliance.

### Notfallversorgung

Eine pädiatrische Notfallversorgung mit Möglichkeiten zur Diagnostik und Therapie, beispielsweise von Flüssigkeits- und Elektrolyt-Verschiebungen sowie anderen metabolischen Störungen sollte für Patient*innen unter Therapie mit Teduglutid rund um die Uhr zur Verfügung stehen. Handelt es sich bei den nächstgelegenen medizinischen Einrichtungen für Notfälle nicht um das primär therapiebegleitende multidisziplinäre Team selbst, muss im Vorfeld festgelegt werden, wie die Kontaktaufnahme zu diesem Team erfolgt.

Das Vorgehen in derartigen Notfallsituationen ist vor Behandlungsbeginn mit den Patient*innen bzw. der Familie abzusprechen.

### Schwangerschaft und Stillzeit

Es liegen bisher keine Erfahrungen mit der Anwendung von Revestive bei Schwangeren vor. Tierexperimentelle Studien ergaben jedoch keine Hinweise auf direkte oder indirekte gesundheitsschädliche Wirkungen im Hinblick auf eine Reproduktionstoxizität. Aus Vorsichtsgründen sollte Revestive jedoch während der Schwangerschaft nicht angewendet werden.

Es ist nicht bekannt, ob Teduglutid in die Muttermilch übergeht. Nach einer einmaligen subkutanen Injektion von 25mg/kg lag die mittlere Teduglutid-Konzentration in der Milch von Ratten bei weniger als 3% der mütterlichen Teduglutid-Plasmakonzentration. Ein Risiko für das gestillte Neugeborene bzw. den Säugling kann nicht ausgeschlossen werden. Aus Vorsichtsgründen sollte Revestive daher während der Stillzeit nicht angewendet werden.

Insbesondere Jugendliche bzw. Patient*innen im Transitionsprozess sind hierauf hinzuweisen.

### Interferenz mit anderen Medikamenten

Es wurden keine Studien zur Erfassung pharmakokinetischer Wechselwirkungen durchgeführt. Eine In-vitro-Studie hat jedoch gezeigt, dass Teduglutid die wirkstoffabbauenden Cytochrom-P450-Enzyme nicht hemmt. Basierend auf der pharmakodynamischen Wirkung von Teduglutid besteht die Möglichkeit einer erhöhten Resorption oral bzw. enteral verabreichter Medikamente.

### Antikörperbildung gegenüber Teduglutid

Bei 33,3% der Patient*innen wurden nach 36 Wochen Teduglutid-Antikörper beobachtet, was bis zur 72. Woche auf diesem Niveau blieb 11 . Bei einigen Patient*innen waren diese Antikörper neutralisierend und haben somit das Potenzial, die Wirkung von Teduglutid zu antagonisieren. Dies wurde nach 72 Wochen bei 10% der Patient*innen berichtet 11 .

### Dokumentation und Qualitätssicherung

Um den Therapie-Effekt objektivieren und bewerten zu können, wird empfohlen, die ambulante Behandlung des Therapieverlaufs zu dokumentieren (siehe Infobox sowie Tab. 1 , Tab. 2 ). Hierbei unterstützend kann auch die Datenerhebung (z.B. Gewicht, Größe, Ausscheidungen) durch einen Homecare-Service erfolgen.

**Table:** **Tab. 1** Empfohlene Untersuchungen vor Behandlungsbeginn.

**Hauptuntersuchungen:**		
**1. Anamnese:** (*fakultativ)		Erfassung der kompletten medizinischen Vorgeschichte mit Dokumentation der Indikationen und Kontraindikationen (Gegenanzeigen) Aktuelle Medikation Lebensqualität*
**2. Gastro/Koloskopie/Sigmoidoskopie:**		Ausschluss eines intestinalen Passage-Hindernisses, von Malignomen und Polypen. Kinder und Jugendliche sollen vor Behandlungsbeginn endoskopiert werden, um anatomische Passagebehinderungen und Malignome auszuschließen, sofern innerhalb der letzten 12 Monate keine solchen Untersuchungen durchgeführt wurden oder innerhalb von 6 Monaten vor Therapiebeginn unerklärliche Blutbeimengungen im Stuhl oder okkultes Blut im Stuhl nachgewiesen wurde.
**3. Radiologie/Sonografie**		Ausschluss von Stenosen und Motilitätsstörungen zur Darstellung bzw. Abklärung der anatomischen und funktionellen Rest-Darmsituation Magen-Darm-Passage (MDP) Ggf. alternativ oder ergänzend MRT Abdomen/Sellink-MRT Leber, intra-/extrahepatische Gallenwege mit Gallenbase, Milzgröße Darmdilatation/Darmmotilität/Darmstenose
**4.** Erfassung **Basisparameter:**		
	a. Status (*fakultativ)	Allgemeinzustand Körperlänge Körpergewicht Kopfumfang bei Kindern unter 2 Jahren Ödeme Blutdruck, Puls Pubertätsstadium Mittlerer Oberarm-Umfang* Hautfaltendicke* Körperzusammensetzung* Erfassung des familiären Risikos für Malignome Tetrazyklin-Unverträglichkeit (Kontraindikation für Revestive) Stuhlfrequenz tagsüber, Anzahl nächtlicher Stuhlgänge, Konsistenz Urin-Ausscheidung (Schätzung von Häufigkeit und Menge)
	b. Blut (*fakultativ)	Elektrolyte, inkl. Mg, Ca, Ph Nierenwerte (Kreatinin, Cystatin C, Harnstoff, Harnsäure) Säure-Basen-Status Leberwerte/Funktionsparameter (Quick, PTT, GOT, GPT, GGT, GLDH, Bilirubin dir./ges., Albumin) Lipase Eisenstatus (Ferritin, Eisen*, löslicher Transferrin-Rezeptor*) Spurenelemente (Zn, Se, Cu) Vitamine (A, E, D, 25-OH-Vitamin-D, Folsäure), Vitamin B12 (Holotranscobalamin*, Methylmalonsäure*) IGF-1, IGFBP-3* Parathormon Aldosteron* Citrullin-Spiegel* (als indirektes Maß der Enterozytenmasse) Testosteron*
	c. Urin	24h-Sammelurin (ggf. Spontanurin) Urin-Elektrolyte (Na, K, Ca, Ph, Mg) Methylmalonsäure/Kreatinin-Quotient* Kreatinin/Eiweiß-Differenzierung Spezifisches Gewicht, Osmolarität
	d. Stuhl (*fakultativ)	Test auf okkultes Blut im Stuhl* Fäkales Calprotectin
	e. Kardiale Untersuchungen	Echokardiografie Elektrokardiogramm (EKG)
	f. Ultraschall	Leber, intra-/extrahepatische Gallenwege mit Gallenbase, Milzgröße, Pankreas Darmdilatation/Darmmotilität/Darmstenose
	g. Ernährungsanalyse, inklusive mind. 3 Tage Ernährungsprotokoll	PE-Volumen PE-Energiegehalt und Zusammensetzung Orale und enterale Makronährstoff- und Flüssigkeitszufuhr
PE = Parenterale Ernährung; Na = Natrium; K = Kalium; Ca = Calcium; Ph = Phosphat; Mg = Magnesium		

**Table:** **Tab. 2** Empfohlene Untersuchungen während der Behandlung.

**Hauptuntersuchungen:**		
**1. Anamnese:** (* fakultativ)		unerwünschte Arzneimittel-Nebenwirkungen, Therapietreue; Lebensqualität*
**2. Gastro-/Koloskopie/ Sigmoidoskopie:**		Bei Blutbeimengungen im Stuhl oder okkultem Blut im Stuhl bei fortgesetzter Behandlung mit Revestive ist für alle Patient*innen eine Kontroll-Endoskopie 1 Jahr nach Therapiestart (Koloskopie oder Sigmoidoskopie) empfohlen, sowie anschließend mindestens alle 5 Jahre (Gastro- und Ileokoloskopie), die insbesondere auch dann zwischenzeitlich erforderlich ist, wenn neue oder ungeklärte gastrointestinale Blutungen bzw. ungeklärte klinische Beschwerden auftreten.
**3. Radiologie/Sonografie** (*fakultativ)		Einmal jährlich Sonografie: Lebergröße, intra-/extrahepatische Gallenwege mit Gallenblase, H.a. Parenchym-Umbau, Splenomegalie, Pankreas Leber-Fibroscan/Elastografie*
**4.** Erfassung **Basisparameter:**		
	a. Status (*fakultativ)	Allgemeinzustand Körperlänge Körpergewicht Kopfumfang bei Kindern unter 2 Jahren Ödeme Blutdruck, Puls Pubertätsstadium Mittlerer Oberarm-Umfang* Hautfaltendicke* Körperzusammensetzung* Stuhlfrequenz tagsüber, Anzahl nächtlicher Stuhlgänge, Konsistenz Urin-Ausscheidung (Schätzung von Häufigkeit und Menge)
	b. Blut (*fakultativ)	Elektrolyte, inkl. Mg, Ca, Ph und Säure-Basen-Status*: in der 1. Woche ca. 1–2x. In der 2./3./4. Woche: je 1x (evtl. durch Kinderfachärzt/in); dann im 1. Halbjahr monatlich. Ab dem 2. Halbjahr vierteljährlich. Darüber hinaus nach Bedarf/Notwendigkeit bzw. im Rahmen der Routine-Diagnostik bei Kindern und Jugendlichen mit chronischem Darmversagen. Nierenwerte (Kreatinin, Cystatin C, Cystatin-C-Clearance, Harnstoff, Harnsäure): monatlich bis 2-monatlich im ersten Halbjahr. Darüber hinaus nach Bedarf/Notwendigkeit bzw. im Rahmen der Routine-Diagnostik bei Kindern und Jugendlichen mit chronischem Darmversagen. Leberwerte/Funktionsparameter (GOT, GPT, GGT, GLDH, Bilirubin dir./ges., Quick/PTT, Albumin): im ersten Halbjahr monatlich bis 2-monatlich. Darüber hinaus nach Bedarf/Notwendigkeit bzw. im Rahmen der Routine-Diagnostik bei Kindern und Jugendlichen mit chronischem Darmversagen. Lipase: im ersten Halbjahr monatlich bis 2-monatlich. Darüber hinaus nach Bedarf/Notwendigkeit bzw. im Rahmen der Routine-Diagnostik bei Kindern und Jugendlichen mit chronischem Darmversagen. Differential-Blutbild, Spurenelemente (Zn, Se, Cu), Vitamine A, E, 25-OH-Vitamin-D, Folsäure, Vitamin B12 (Holotranscobalamin*, Methylmalonsäure*), Eisenparameter: 1–2-mal im ersten Halbjahr. Darüber hinaus nach Bedarf/Notwendigkeit bzw. im Rahmen der Routine-Diagnostik bei Kindern und Jugendlichen mit chronischem Darmversagen. Parathormon: 1x nach 3–6 Monaten, dann nach Bedarf/Notwendigkeit bzw. im Rahmen der Routine-Diagnostik bei Kindern und Jugendlichen mit chronischem Darmversagen Aldosteron*: nur bei Patient*innen mit V.a. sekundären Hyperaldosteronismus Citrullin-Spiegel*: als Verlaufsparameter zur indirekten Messung der Enterozytenmasse einmal jährlich
	c. Urin	24h-Sammelurin/Ausscheidung pro 24h Urin-Elektrolyte (Na, K, Ca, Mg, Ph) (ggf. Spontanurin) Methylmalonsäure/Kreatinin-Quotient* (alternativ zur Bestimmung im Blut) Kreatinin/Eiweiß-Differenzierung Spezifisches Gewicht
	d. Stuhl (*fakultativ)	Test auf Blut im Stuhl: jährlich fäkales Calprotectin: jährlich Bei Nachweis von Blut im Stuhl oder ungeklärten Entzündungshinweisen: Indikation zur Endoskopie zum Ausschluss intestinaler Polypen.
	e. Kardiale Untersuchungen	1 x nach einem Jahr der Behandlung sowie jederzeit bei Hinweis auf kardiale Belastung, Insuffizienz, Flüssigkeitsüberladung oder risikoadaptiert bei neu aufgetretener kardialer Morbidität Echokardiografie EKG
	f. Ernährungsanalyse, inklusive mind. 3 Tage Ernährungsprotokoll	PE-Volumen PE-Energiegehalt und -Zusammensetzung Orale und enterale Makronährstoff- und Flüssigkeitszufuhr
Zn = Zink; Se = Selen; Cu = Kupfer; Na = Natrium; K = Kalium; Ca = Calcium; Mg = Magnesium; Ph = Phosphat		

Die Teilnahme an Registern wird empfohlen, da auf diese Weise wichtige Erkenntnisse aus der Behandlung von Kurzdarm-Patient*innen gewonnen und insbesondere Daten zur Langzeitanwendung mit Teduglutid gesammelt werden können 41 . Studien haben gezeigt, dass die Teilnahme an Registern (z.B. bei chronisch-entzündlichen Darmerkrankungen) zu einer leitliniengerechteren Behandlung führt 42 . Im Rahmen des Forschungsprojekts TEDUREG (DRKS00021006) werden aktuell Daten von Kindern und Jugendlichen im Alter von 1–18 Jahren mit Kurzdarmsyndrom erhoben, die in Deutschland oder Österreich mit Teduglutid behandelt werden. Dabei werden Daten zum Typ des Kurzdarmsyndroms (einschließlich der Restdünndarmlänge), zur parenteralen und enteralen Ernährung vor und während der Therapie mit Teduglutid, sowie zu klinischen Untersuchungsergebnissen (Stuhlgang, Wachstum, Gewicht), Laborwerten (z.B. Leber- und Nierenwerte), Komorbiditäten (z.B. Leber-Erkrankung) und Komplikationen (z.B. Katheter-Infektionen, Polypen) erfasst und analysiert. Ziel ist es, wichtige weitere Erkenntnisse zur Wirksamkeit, zu Begleiteffekten, sowie zur Prognose und Sicherheit der Behandlung im Langzeitverlauf zu gewinnen. Außerdem wurde das Register für Kinder und Erwachsene mit Kurzdarm (REKUDA) ( https://rekuda.dgem.de ) etabliert. Darüber hinaus gibt es das pädiatrische „International Intestinal Failure Registry (IIFR)“ 41 und das globale, prospektive „Registry for Participants with Short Bowel Syndrome (TED-R13–002)“ (NCT01990040, EUPAS7973).

### Monitoring

Die bei Behandlungsbeginn empfohlenen Untersuchungen sind in Tab. 1 aufgeführt; die im Verlauf empfohlenen Untersuchungen in Tab. 2 . Sie ergeben sich sowohl aus dem allgemeinen Monitoring bei heimparenteraler Ernährung 43 sowie aus den spezifisch berichteten Effekten und Nebenwirkungen der Behandlung mit Teduglutid 11 12 . Die regelmäßige Überwachung, die eine körperliche Untersuchung, die Messung der Körpermaße, sowie die Kontrolle der Urin- und Stuhlausscheidung und der laborchemischen Werte umfasst, dient der Erkennung von Elektrolyt- und metabolischen Störungen sowie der Steuerung der Volumen- und Nährstoff-Zufuhr. Bei jeder ambulanten Verlaufskontrolle wird die parenterale Ernährung dementsprechend überprüft, ggf. neu kalkuliert und die Rezeptur angepasst. Die Entscheidung über Änderungen der Nahrungs- und Flüssigkeitszufuhr liegt bei den behandelnden Ärzt*innen. Maßgebend für eine Änderung der parenteralen Ernährung unter Teduglutid sind ein Ungleichgewicht bei der oralen Flüssigkeitszufuhr und der Urinausscheidung (z.B. ±400ml/m ^2^ ) sowie der zu erwartende, altersentsprechende Gewichtsverlauf (z.B. ±5%) 9 . Nach jeder Änderung in der Kalorien-, Flüssigkeits- oder Elektrolytzufuhr der PE ist eine Verlaufskontrolle erforderlich.

Ein Anstieg von Hämoglobin und Citrullin in den ersten 6 Monaten der Behandlung war in der europäischen, multizentrischen Kohortenstudie ein Prädiktor für eine vollständige PE-Entwöhnung 24 .

### Beendigung der Therapie

In der Fachinformation von Revestive wird das Ausbleiben einer „allgemeinen Verbesserung des Zustandes des Patienten“ als Grund für die Beendigung der Behandlung mit Teduglutid genannt. In den Studien wurde gezeigt, dass die Effekte einer Teduglutid-Behandlung auch noch nach den ersten 12 bzw. 24 Wochen im ersten Jahr der Behandlung auftreten 20 22 .

Wird unter der Behandlung mit Teduglutid eine enterale Autonomie erreicht, ist eine Reduktion oder Beendigung der Behandlung zu erwägen. Gerade im Kindesalter kann im Rahmen der Adaptationsprozesse im Einzelfall eine anhaltende enterale Autonomie ohne Fortführung der Therapie mit Teduglutid erreicht werden. Daten zum optimalen Zeitpunkt, den Erfolgschancen und dem konkreten Vorgehen hierzu liegen jedoch derzeit nicht vor.

## Schlussfolgerung

Trotz der Behandlungskosten und der in der Therapievorbereitung und -kontrolle aufzubringenden Ressourcen besteht aufgrund der zunehmenden Evidenz zur Wirksamkeit und Sicherheit derzeit ein Konsens, der die Anwendung von Teduglutid bei Patient*innen mit KDS und chronischem Darmversagen auch im Säuglings-, Kindes- und Jugendalter im Gesamtkontext einer multidisziplinären Versorgung befürwortet. Klinische Studien bei Kindern mit KDS haben die Wirksamkeit zur Reduktion der parenteralen Ernährung bestätigt. Es fehlen jedoch noch weitere Daten, um den individuellen Nutzen, den optimalen Therapiebeginn und die Dauer der Therapie sowie Nebenwirkungen unter Langzeitbehandlung vorherzusagen.

### Glossar (gemäß 3 )

- Chronisches Darmversagen (CDV): Kritische Verringerung der Darmmasse oder Darmfunktion unter das Minimum, das für die Absorption der für ein adäquates Wachstum erforderlichen Nährstoffe und Flüssigkeiten bei Kindern notwendig ist, und zwar über einen Zeitraum von mindestens 60 Tagen innerhalb von 74 aufeinanderfolgenden Tagen.
- Kurzdarmsyndrom (KDS): die Folge eines natürlichen Verlustes oder einer chirurgischen Resektion des Dünndarms (KD).
- KDS-Darmversagen (KDS-CDV): KDS, bei dem der kritische Wert für die Nährstoffversorgung und ein angemessenes Wachstum unterschritten wird.
- Parenterale Ernährung (PE): intravenöse Verabreichung von Wasser, Makronährstoffen (Glukose, Fette, Aminosäuren), Elektrolyten, Mineralien und Mikronährstoffen (Spurenelemente und Vitamine).
- Häusliche parenterale Ernährung (HPE): Verabreichung von PE in häuslicher Umgebung außerhalb des Krankenhauses bei klinisch stabilen Patient*innen, die voraussichtlich für mindestens 3 weitere Monate PE benötigen und bei denen die Eltern/Betreuer ein strukturiertes Schulungsprogramm absolviert haben.
- Enterale Ernährung (EN): Die Verabreichung von Nährstoffen über den Magen-Darm-Trakt kann unterteilt werden in orale Ernährung und medizinische Ernährungstherapie über eine Ernährungssonde (nasogastrische Sonde, nasojejunale Sonde, Gastrostomie oder Jejunostomie). Die Definition der Deutschen Gesellschaft für Ernährung in der Medizin (DGEM) unterscheidet sich hiervon und bezieht enterale Ernährung ausschließlich auf die Verabreichung von Nährstoffen über Ernährungssonden in den Magen-Darm-Trakt 44 . Die Ernährung mit Sondensystemen kann weiter unterteilt werden in kontinuierliche, intermittierende oder Bolusfütterung.

## Notes

Empfehlung 1: Behandlungsindikation I. Bei Kindern und Jugendlichen mit Kurzdarmsyndrom soll eine Therapie mit Teduglutid in Betracht gezogen werden, wenn die intestinale Adaptation trotz optimierter individueller Behandlungsmaßnahmen stagniert (Evidenzgrad 2, Empfehlungsgrad A, starker Konsens 100%). II. Eine individuelle Nutzen-Risiko-Bewertung soll den Nutzen der Teduglutid-Behandlung für Patient*innen höher als mögliche Risiken bzw. Nebenwirkungen bewerten (Evidenzgrad 4, Empfehlungsgrad A, starker Konsens 100%). III. Eine Behandlung mit Teduglutid soll nicht anstatt einer multidisziplinären Betreuung durch eine für das Kurzdarmsyndrom erfahrene Kinder-Gastroenterologie erfolgen (Evidenzgrad 4, Empfehlungsgrad A, Konsens 83%).

## Notes

Empfehlung 2: Aufklärung und Betreuung I. Die Indikationsstellung, Aufklärung und Behandlung soll durch eine mit dem Kurzdarmsyndrom erfahrene pädiatrische Gastroenterologie bzw. nach Rücksprache mit einer/einem bei Kurzdarmsyndrom erfahrenen Kollegin/Kollegen bzw. einem Zentrum für intestinale Rehabilitation erfolgen (Evidenzgrad 5, Empfehlungsgrad A, starker Konsens 100%). II. Kinder mit KDS, die eine heimparenterale Ernährung bei Darmversagen erhalten und mit Teduglutid behandelt werden, sollten von einem mit der Behandlung erfahrenen multidisziplinären Team überwacht werden (Evidenzgrad 1, Empfehlungsgrad B, starker Konsens 100%).

## Notes

Empfehlung 3: Therapiebeginn I. Nach dem letzten stattgehabten chirurgischen Eingriff sollte ein angemessener Zeitraum zur intestinalen Adaptation und für einen Kostaufbau von circa 3–6 Monaten abgewartet werden (Evidenzgrad 4, Empfehlungsgrad B, Konsens 92%). II. Es sollte eine metabolisch stabile Phase des chronischen Darmversagens vorliegen und es konnte die für ein perzentilenparalleles Gedeihen erforderliche parenterale Nährstoff- und Flüssigkeitszufuhr in den letzten 3 Monaten nicht weiter reduziert werden (Evidenzgrad 4, Empfehlungsgrad B, starker Konsens 100%). III. Eine patientenbezogene Diagnostik und Therapie in Bezug auf z.B. Ernährung, intestinale Entzündung, Transportstörung, bakterielle Fehlbesiedelung oder Restdarm-Anatomie sollte erfolgt sein (Evidenzgrad 5, Empfehlungsgrad B, Konsens 83%). IV. Nach interdisziplinärem Austausch sollte sich keine Indikation für eine chirurgische Behandlung (z.B. durch Wiederherstellung einer Darmkontinuität oder eine Darmverlängerungsoperation) oder eine andere Optimierung im Management des Kurzdarmsyndroms ergeben haben (Evidenzgrad 4, Empfehlungsgrad B, starker Konsens 100%). V. Eine Steigerung der oralen/enteralen Ernährung soll möglich sein, damit das Therapieziel der Reduktion der parenteralen Ernährung und der infusionsfreien Tage erreicht werden kann. Diesbezüglich sollten relevantes Erbrechen, Motilitätsstörungen, Kapazitätsprobleme oder Stenosen nicht vorliegen. Eine Ernährungsanalyse und -Beratung soll durch eine mit dem Kurzdarmsyndrom erfahrene Ernährungsfachkraft stattgefunden haben (Evidenzgrad 4, Empfehlungsgrad A, Konsens 92%). VI. Die Nachsorge nach Therapiebeginn zur Steuerung der parenteralen, enteralen und oralen Ernährung, eine Labordiagnostik, das Erkennen von Nebenwirkungen und eine Langzeit-Überwachung inklusive Endoskopie sollten sichergestellt sein (Evidenzgrad 5, Empfehlungsgrad A, starker Konsens 100%). VII. Gegenanzeigen für die Anwendung von Teduglutid sollen ausgeschlossen sein: bekannte Überempfindlichkeit gegen Inhaltsstoffe; aktive oder vermutete Malignität bzw. zurückliegende Malignität (in den vergangenen 5 Jahren) im Bereich des Gastrointestinaltrakts, einschließlich des hepatobiliären Systems und des Pankreas (Evidenzgrad 4, Empfehlungsgrad A, starker Konsens 100%). VIII. Relevante kardiale Erkrankungen sollen ausgeschlossen werden (z.B. hämodynamisch relevante Herzfehler, Non-Compaction-Kardiomyopathie) (Evidenzgrad 4, Empfehlungsgrad A, starker Konsens 100%). IX. Die Zusammenarbeit zwischen behandelnden Ärzt*innen und Patient*innen bzw. deren Sorgeberechtigten sollte von einer guten Adhärenz hinsichtlich des Behandlungsprozesses und der Therapieziele geprägt sein, weil dies den Behandlungserfolg fördert. Außerdem sollte bei Patient*innen eine gute Akzeptanz bezüglich der subkutanen Gabe von Teduglutid bestehen (Evidenzgrad 5, Empfehlungsgrad B, starker Konsens 100%). X. Im Rahmen von kollegialen Falldiskussionen können Indikation und Therapie erörtert werden. Diese dienen sowohl der Qualitätssicherung als auch der Dokumentation (Evidenzgrad 5, Empfehlungsgrad C, starker Konsens 100%).

## Notes

Infobox – Dokumentation

- Erfassung und Meldung unerwünschter Ereignisse: durch Ärzt*innen bzw. Pflegepersonal (gesetzlich vorgeschrieben)
- Patiententagebuch (dauerhaft)
- Kindergarten-/Schulfehltage: Dokumentation durch Sorgeberechtigte
- Therapietreue: Standardabfrage und Dokumentation bei jedem Patientenkontakt
- Lebensqualität (fakultativ): max. alle 6 Monate durch Sorgeberechtigte und Kind

## Notes

Empfehlung 4: Überwachung I. Eine regelmäßige wöchentliche Überwachung, einschließlich Gewicht, Urinabgang, Blutharnstoff und Elektrolyte, sollte für mindestens 4 Wochen nach Beginn der Behandlung mit Teduglutid sowie eine Woche nach jeder Änderung der infundierten PE-Zusammensetzung erfolgen (Evidenzgrad 3, Empfehlungsgrad B, Konsens 92%). II. Mittel- und langfristige Kontrolluntersuchungen sollten erfolgen, um unerwünschte Nebenwirkungen, auch im Langzeitverlauf zu identifizieren oder die (parenterale) Ernährung zur Vermeidung von Nährstoff-Defiziten bzw. -Überangeboten anzupassen, sowie den Therapie-Erfolg bzw. das Therapieversagen zu evaluieren (Evidenzgrad 3, Empfehlungsgrad B, starker Konsens 100%). III. Bei ausbleibendem Therapie-Ansprechen innerhalb der ersten 12 Monate nach Therapiebeginn sollte die Beendigung der Teduglutid-Anwendung erwogen werden (Evidenzgrad 5, Empfehlungsgrad B, starker Konsens 100%).
